# Evaluation of smartphone APP-based case-management services among antiretroviral treatment-naïve HIV-positive men who have sex with men: a randomized controlled trial protocol

**DOI:** 10.1186/s12889-020-8171-5

**Published:** 2020-01-20

**Authors:** Xiaoyan Fan, Rui She, Cong Liu, Haidan Zhong, Joseph T. F. Lau, Chun Hao, Jinghua Li, Yuantao Hao, Linghua Li, Jing Gu

**Affiliations:** 1 Department of Medical Statistics, School of Public Health, Sun Yat-sen University, Guangzhou, 510080 Guangdong People’s Republic of China; 2 Centre for Health Behaviours Research, School of Public Health and Primary Care, Faculty of Medicine, The Chinese University of Hong Kong, Hong Kong, People’s Republic of China; 3 Infectious Disease Centre, Guangzhou Eighth People’s Hospital, Guangzhou, Guangdong People’s Republic of China; 4 Centre for Medical Anthropology and Behavioural Health, Sun Yat-sen University, Guangzhou, Guangdong People’s Republic of China; 5 Sun Yat-sen Global Health Institute, Sun Yat-sen University, Guangzhou, Guangdong People’s Republic of China

**Keywords:** HIV, Men who have sex with men, mHealth, Case management, Adherence

## Abstract

**Background:**

Men who have sex with men (MSM) are disproportionally affected by HIV in China. ‘Treatment as Prevention’ is a promising strategy for HIV prevention but requires adequate adherence. Mobile health (mHealth) may be an acceptable and feasible approach for service delivery, but there is little evidence supporting mHealth intervention for improving antiretroviral treatment adherence among HIV-infected MSM in low- and middle-income countries, including China. This study will aim to develop a smartphone application-based case-management service and compare its efficacy to standard care with regards to adherence, CD4, HIV viral load and psychosocial outcomes among MSM patients in Guangzhou, China.

**Methods:**

A non-blinded 1:1 parallel-group randomised controlled trial will be conducted in Guangzhou Eighth People’s Hospital, with 300 MSM enrolled in each arm. Eligible MSM who are newly initiating ART will be randomly assigned to an intervention group (standard-of-care case management plus mHealth intervention) or a control group (standard-of-care case management). The development of the mHealth intervention will be based on the information–motivation–behavioural skills theory of ART adherence, and comprise four components: educational articles, one-to-one online communication with case managers, support-service information and hospital-visit reminders. Outcome measures will be collected at baseline and at months 1, 3, 6, and 12. The primary outcomes will be ART adherence and CD4 count at month 6. Secondary outcomes include HIV RNA, sexual behaviours, mental health status, illness perceptions, and quality of life. *χ*^*2*^ test and t-test will be used for between-group comparisons. Intervention effects will be evaluated using General estimating equation performed by SAS 9.0, on the principle of intention-to-treat. Structural equation modelling will be used to test potential mechanisms of intervention effect.

**Discussion:**

This study is the first to explore the efficacy of mHealth intervention in the case management services targeted at HIV-infected MSM in low-and middle-income countries. Once proven effective, the innovative mHealth service could be integrated into the routine case management of PLWH. as well as be tailored to the patient management service for other chronic conditions.

**Trial registration:**

ClinicalTrial.gov: NCT03860116; Registered on 1 March 2019.

## Background

Men who have sex with men (MSM) have been identified as one of the key populations in the HIV epidemic. The prevalence of HIV among MSM in China has been rapidly and continuously increasing, especially in metropolitan cities such as Beijing, Guangzhou, and Chengdu [1–3]. The pooled prevalence of HIV infection among MSM soared from 1.4% in 2001 to 9.0% in 2013 [4], and transmissions between MSM accounted for 25.5% of the new HIV infections in 2017 [5].

‘Treatment as Prevention’ (TasP) is a promising strategy for HIV prevention and control. In 2016, the Chinese Centre for Disease Control and Prevention (CDC) revised the national guidelines for antiretroviral treatment (ART) and suggested immediate treatment for all people living with HIV/AIDS (PLWHA) [6]. However, the long-term success of such efforts to scale-up coverage of ART for PLWHA may be negatively affected by deficits in adherence to the spectrum of HIV care. ART requires adequate adherence (usually defined as intake of at least 95% of ART doses [7, 8]) to yield satisfactory clinical outcomes, e.g., suppressing HIV viral replication [9], improving quality of life [10], and preventing transmission [11]. However, a recent meta-analysis indicated that only 77.6% (95% confidence interval [CI]: 71.6–83.1) of Chinese PLWHA on ART maintained adequate adherence [12]. Barriers to ART adherence include patient factors (e.g., education, lack of self-efficacy, mental illness), medication factors (e.g., side effects, pill burden, and food requirement), and system of care (e.g., linkage of care, negative experiences of healthcare system) [13–15]. Moreover, MSM can experience a fear of disclosure in public and healthcare settings and the stigma associated with their sexual orientation [16]. Thus, the ART adherence of this group warrants more attention.

Case management has been regarded as a routine practice to address the complex medical barriers to and needs for ART adherence [17, 18]. It incorporates a wide range of medical and supportive services and is routinely used in many countries. It has been shown to be effective in increasing CD4 counts [19], and in improving care engagement, ART adherence and quality of life [20, 21], as well as in reducing transmission-risk behaviours among PLWHA [22]. Concurrently, the development of information technology has led to a paradigm of mobile health (mHealth), which is characterized by the communication and delivery of health services via text messages, smartphone applications (APPs), websites, and social media. Consequently, mHealth has become a highly accessible and adaptive approach to medical practice, especially in low- and middle-income countries [23]. Currently, smartphones are used by 68% of the population in China [24], with up to 97.5% of MSM having a smartphone [25]. Adherence-supporting interventions incorporating mHealth have shown good acceptability and feasibility in HIV-positive MSM [26], and demonstrated efficacy in improving PLWHA’s linkage to care, retention in treatment and adherence to ART [27, 28]. However, to the authors’ knowledge, mHealth interventions to improve the adherence of HIV-positive MSM to ART and potential mechanisms of how an intervention works have not been fully explored in China, and many mHealth interventions have mainly relied on text messages [29]. Thus, there is a need for a rigorously designed and comprehensive mHealth intervention tailored to Chinese HIV-positive MSM.

**Fig. 1:**
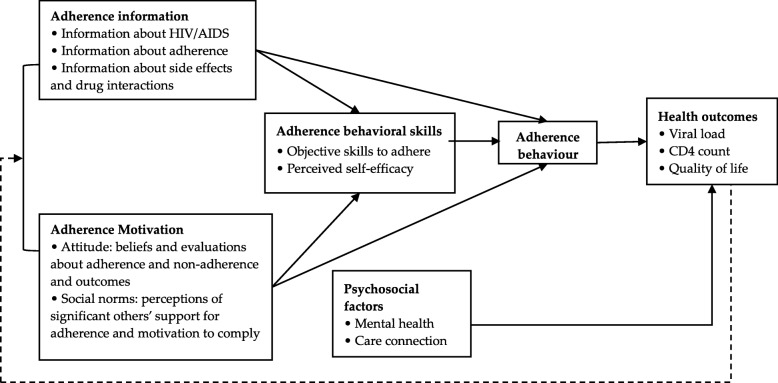
Theoretical framework

In addition, it has been suggested in reviews that theory-based behavioural interventions are more likely to be effective than non-theory-based interventions [30]. The Information-Motivation-Behavioural Skills (IMB) model of ART adherence, for example, is widely used to understand the dynamics of ART adherence and to intervene with patients to promote better adherence [31]. This model has been effectively tested across diverse populations worldwide [32–35] and has often been used to guide the development of interventions to promote HIV-related healthy behaviour, such as ART adherence, retention and safe sex [36–39]. According to the IMB model, information on HIV and ART interacts with an individual’s motivation to take ART medication, thereby influencing the development of behavioural skills related to adherence; in other words, well-informed and motivated patients with adequate skills for enacting adherence-related behaviours will exhibit better adherence [40]. Further, psychological functioning (e.g., depression) and access to care are moderating factors in the associations between predictors and adherence outcomes [31] (Fig. 1).

The objective of this study is to design and evaluate the efficacy of an APP-based case-management intervention among MSM who are newly initiating ART, using the IMB model as the theoretical framework. We have trained nurses to deliver the intervention, and here we describe the protocol of what will be an ongoing open-label randomised controlled trial. The SPIRIT guidelines were adhered to for reporting this manuscript.

## Methods

### Study aim and design

This is a single-centre, parallel-group, open-label randomised controlled trial. We have designed a comprehensive smartphone APP-based case-management service model for MSM newly initiating ART, and will evaluate its efficacy compared to standard-of-care (SOC) case-management services in improving ART medication adherence, HIV clinical indicators, and psychosocial outcomes. The collaborative technology company Trusted Doctor Inc. has developed a smartphone APP (‘Trusted Doctor’) that is linked to WeChat. WeChat is the most popular social media platform in China and has more than 1.1 billion active users according to Tencent Inc.’s 2019 annual report; 93% of the residents in the major cities of China use WeChat every day [41].

This study will be based on the combination of ‘Trusted Doctor’ and WeChat by establishing a link, called *WeChat official account*, between the two smartphone APPs. Certified health care providers (HCPs; i.e., doctors, nurses, or case managers) can use the ‘Trusted Doctor’ APP to have direct and instantaneous online interactions with patients. And patients can use the WeChat platform to access their health care providers, as well as receive services including article alerts, follow-up questionnaires, appointment reminders, free phone calls, etc.

### Study setting

The study is being conducted in Guangzhou, the capital city of Guangdong Province in the southeast of China. HIV prevalence among MSM in Guangzhou increased from 3.9% in 2009 to 11.4% in 2013 [42]. By the end of 2018, 10,453 HIV cases had been reported in Guangzhou and 9161 PLWHA (87.6%) were undergoing ART (data extracted from China’s National Information System of HIV/AIDS prevention and treatment). Participant recruitment and implementation of this study is in cooperation with Guangzhou Eighth People’s hospital, the first authoritative hospital for ART provision in Guangdong. The hospital has provided > 18,000 PLWHA from all around the country with ART and follow-up supportive services and has been exploring the use of case-management services for PLWHA since 2018, in the form of educational sessions and periodic physical visits. Supportive counselling is also provided, if necessary.

### Participants

#### Eligibility criteria

The participant inclusion criteria are as follows: 1) male, aged ≥18; 2) HIV-positive; 3) ART-naïve, and planning to initiate ART on the day of recruitment; 4) self-reported to be HIV-infected through homosexual transmission; 5) having access to the Internet on a smartphone; 6) having a WeChat account and using it in daily communication; 7) willing to provide written informed consent. Participants who have been hospitalized due to severe opportunistic infections are excluded.

#### Recruitment, enrolment and randomization

Potential participants are being recruited from the collaborative hospital that provides ART for PLWHA. Four nurse case managers with previous experience of case management are responsible for participant recruitment and intervention delivery. Before the commencement of this study, all nurse managers received two four-hour training sessions on recruitment, data collection, intervention specification, and tutoring about the APP. After nurse case managers confirm their eligibility and obtaining written informed consent, participants are invited to complete baseline assessments and then randomly assigned to intervention (SOC + mHealth) or control groups (SOC) in a 1:1 allocation ratio. Block randomization (block length = 4) is adopted and random numbers are generated with SAS 9.4.

Written records of group-assignments are sealed in individual opaque envelopes marked with study identification numbers. The intervention group then receive brief training in use of the WeChat program official account from the investigators and are reminded to not forward the WeChat platform to others, to avoid potential confounding effects. Both the control and intervention group are required to ‘friend’ the research team’s WeChat account for data collection and to provide user feedback. Participants’ enrolment information including name, research ID (RID), medical ID, phone number, and their case managers, are documented. The research ID is composed of a skewed date (month plus one) and the sequence of the intraday enrolment to ensure confidentiality. All participants are encouraged to refrain from seeking other health interventions during the active study period. Standard operating procedure (SOP) and program manuals have been developed to guide the study.

### Intervention

#### Control group

The control group receives SOC service at the hospital, which commences with a 20-min ART education session for MSM patients newly initiating ART. Follow-up hospital visits are then arranged at one, two, three and 6 months after ART initiation. During each hospital visit, patients undergo physical assessment, receive prescription refills, meet with case managers and schedule the date of their next appointment. Case managers track patients’ medication adherence, sexual behaviours, mental health, sleep quality and weight changes to confirm that the current treatment regimen is suitable. Supportive counselling will be provided to patients, if necessary. If the patients display adequate adherence in the initial three-month period, the refill interval will be extended to 3 months, and case managers will then schedule a fourth meeting at 6 months. After that, patients with stable medication-taking and hospital-visiting behaviour will be referred to the non-governmental organization (NGO) *Red Ribbon* for scheduling of prescription refills.

#### Intervention development

The intervention group receive SOC service and additional mHealth interventions. The design of the mHealth intervention is ground in and informed by the IMB model, which is the most widely used conceptual model for ART adherence. The core content of effective interventions must address the underlying determinants of ART adherence, which, according to the IMB model, include access to adherence-related information, motivation, and behavioural skills.

Elicitation research was conducted to clarify the information, motivation, and behavioural factors that underlie adherence or nonadherence in the targeted group. This involved an extensive review of the literature, lengthy conversations with experts in the field, and elicitation work with multiple stakeholders, including ten HIV-infected MSM with different durations of ART, four nurse case managers, three software engineers, and four NGO staff. These inputs were used to tailor the intervention design, delivery, and content to the needs and characteristics of HIV/AIDS patient, and identified four major intervention components: exposure to educational articles, online communication, supportive service information and hospital-visit reminders (Table 1).

**Table 1:** Innovative case management service model

Case management	IMB constructs	Service content	Intervention components
Assessment	Motivation	Establishing rapport	Face-to-face communication
Plan	Motivation	Discussing treatment plan	Face-to-face communication
Service	Information	Knowledge regarding HIV/ART	Educational articles
	Information	Knowledge regarding treatment process?	Educational articles
	Information	Information about preventing transmission	Educational articles
	Behavioural skills	Behavioural skills of adherence	Face-to-face/Online communication
	Behavioural skills	How to promote partner testing	Face-to-face/Online communication
	Behavioural skills	Avoidance of risky behaviour	Face-to-face/Online communication
	Mental health	Counselling	Face-to-face/Online communication
	Care connection	STI treatment	Supportive service information
Coordination	Mental health	Psychological support	Supportive service information
	Care connection	Abstinence therapy	Supportive service information
	Care connection	Other social service	Supportive service information
Evaluation	Care connection	Tests results retrieval	Supportive service information
	Care connection	Follow-up service	Hospital visit reminders

##### Delivery of educational articles

According to the IMB model, if a patient possesses the requisite adherence information, they are more likely to enact specific adherence behavioural skills and exhibit behavioural change. Thus, during the 6 months of the intervention period, a series of educational articles containing ART-adherence information and adherence skills are delivered automatically and freely to the participants via the WeChat official account.

The content of the educational articles was developed based on a literature review, individual interviews, and group discussions. For instance, qualitative interviews of ART-experienced MSM were conducted to discuss the health concerns and information they needed most during their initial 6 months of ART. According to the needs expressed by interviewees, different topics of educational articles were derived, and approximately 1400 educational articles were subsequently collected from social media accounts. Six trained medical undergraduates worked in pairs to read and screen these articles for comprehensibility and timeliness. The final selection of 209 articles was authorized by the authors and then edited to add illustrations and remove sensitive words (e.g. HIV/AIDS) from the titles to avoid inadvertent disclosure of HIV status. Then, the modified articles were examined by physicians to ensure the information was valid and timely. The articles spanned 13 topics related to aspects such as adherence, side effects, and mental health. Detailed information on the contents of the articles is shown in Table 2.

**Table 2:** Educational article topics

Topic of articles	Numbers	Topic of articles	Numbers
Adherence	6	Laws and regulations	19
Basics about HIV and ART	14	Mental health	10
Comorbidities	33	Notes about medication taking	10
Daily life	46	Other concerns	5
Disclosure	4	Side effects	19
Drug resistance	8	Transmission prevention	9
Interpretation of test results	14	–	12

To improve the accessibility and effectiveness of the intervention, the time and frequency for supplying information to participants was considered. In terms of timing, it was decided to send the educational material to participants at 10 p.m. to optimize subjects’ assimilation of the information. This timing was for three reasons: 1) a previous study had suggested that the rate of reading articles published on WeChat accounts peaked at 10.00 p.m. [43]; 2) individual MSM patients had suggested in interviews that educational articles should be delivered at night to avoid inadvertent sero-status disclosure to colleagues in the daytime or to friends during off hours. 3) case managers also recommended 10:00 p.m. for article-delivery, as this would be close to most patients’ daily medication-taking schedule, i.e., half an hour before patients’ bedtime.

The frequency of articles delivered are different over time to avoid inciting negative behaviours among the participants [44]. Thus, in the first 2 weeks of month one, educational materials are sent to the participants daily, and then for the second 2 weeks of month one, they are sent three times per week. After this, two articles are delivered per week in the following 2 weeks, and then once per week for the rest of the intervention period. Currently, 132 participants have been assigned to the intervention group and a total of 44 articles have been sent to this arm, with an average of 112 reading times for each article. Participants can also retrieve previous published articles at any time, with instructions on retrieval reiterated in monthly reminders.

##### Individual online communication

One-to-one online communication can be initiated by participants or nurses to provide social and instrumental support during the process of ART. Participants can send messages to the nurse case managers if they have any question regarding ART, and nurses are requested to promptly respond to the inquiries, i.e., within 2 days. In addition, automatic messages are sent to each participant asking about their medication-taking behaviours and mental health status at day 7 and day 14 after ART initiation, which were suggested by case managers as being important time points for identification of non-adherent behaviour and to improve adherence.

The purpose of these nurse–participant interactions is to evaluate participants’ progress and help identify barriers to progress, and nurses are required to provide tailored feedback and personalized information to an enquiring patient. If a patient expresses negative emotions during the individual online communication, the case managers will, if necessary, provide emotional comfort and counselling. Considering the feasibility of intervention, the numbers of messages that patients can send are limited to a certain level to balance the workload of case managers and the needs of patients.

##### Hospital-visit reminders

A message-reminder module for hospital visits was designed to run autonomously, to facilitate patients’ medication-taking and retention in the ART program. Four follow-up hospital visits are scheduled at months 1, 2, 3 and 6 after ART initiation. Case managers and patients discuss and agree on an exact date for each hospital visit at patients’ convenience. The reminder messages are sent 7 days prior to each scheduled hospital visit. The wording of the reminders is intentionally vague to avoid unwanted disclosure of HIV infection.

##### Supportive service information

The IMB model suggests that psychological functioning (e.g., depression) and access to care might moderate the associations between predictors and adherence outcomes [31]. Thus, in this study we provide easily accessible linkage and referrals to supportive services, including psychological counselling, sexually transmitted diseases (STD) treatment, detoxification, LGBT support (from an NGO), amongst others. Interviews with staff of psychological institutes, NGOs, and detoxification service centres revealed major variations in services provided by different organizations in terms of cost and approach, and the complexity that would arise if those services were all to be provided in our program.

**Fig. 2:**
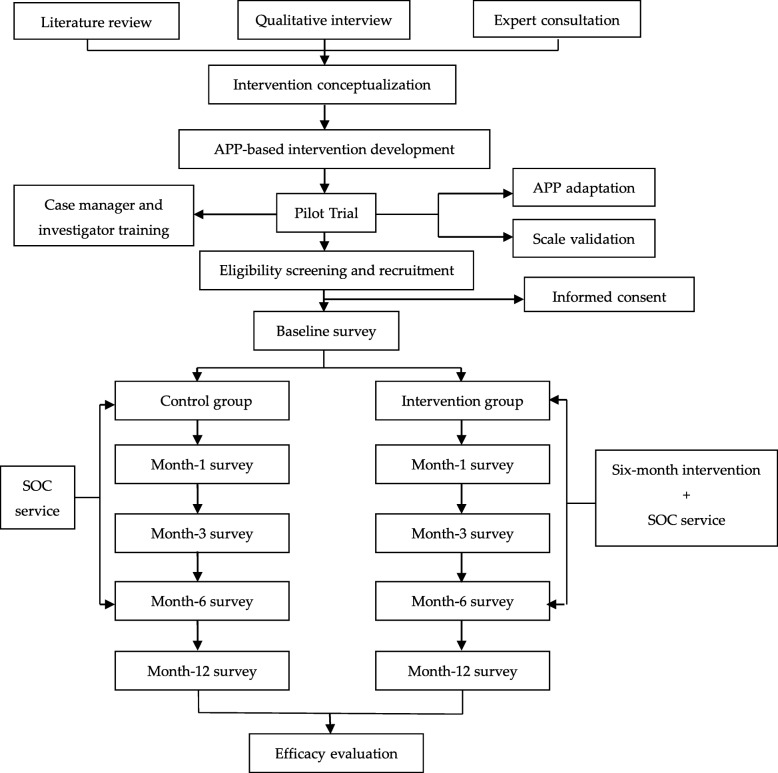
Flow chart of the trial

Therefore, information regarding the location, opening hours, and potential charges of these supportive service institutions are collated and sent to patients monthly via WeChat, as a module of interventions delivered in the present study. Patients can also easily require services from relevant organizations using the WeChat platform. The flow chart is shown in Fig. 2.

### Pilot study

A 1-month pilot study was conducted among 20 participants to assess the feasibility and acceptance of intervention, and detect potential problems in wording or length of the questionnaire, thus providing information that might help to optimize the design of the intervention. Thus, some questionnaire wording was revised after the pilot study, and the limit on the number of messages that could be sent by patients was adjusted according to patients’ feedback. During the initial pilot phase, the nurse case managers were observed and supervised in the whole process of recruitment and implementation, and were advised if they displayed any inappropriate expressions or behaviours, e.g., exaggerating the benefits of the intervention or recommending other health information resources to participants. An additional training session were delivered to case managers to strengthen the SOP after the pilot study.

### Patient and public involvement (PPI)

PPI representatives worked with us to develop and refine the design of the intervention. However, it was difficult to involve patients in other areas of the study due to data protection restrictions. Patients were not consulted to develop patient-relevant outcomes or interpret the results. Patients were not invited to contribute to the writing or editing of this document for readability or accuracy.

### Data collection

Baseline assessment is initially being conducted at the enrolment procedure on a tablet, under the instruction of investigators. Data on demographic characteristics (age, marital status, education, monthly income, employment, and eHealth literacy), HIV status (transmission route, duration after diagnosis, sexual orientation, sero-status disclosure, STDs), psycho-social conditions (mental health, quality of life, illness representation, perceived stigma, social support, IMB constructs) and risky behaviours (sexual and drug use behaviours) are collected.

Follow-up assessments will be scheduled at months 1, 3, 6 and 12 (6-month post-intervention). The content of follow-up assessments at each time-point is described in Table 3, and covers variables related to ART adherence, mental health, illness representation, sexual risk behaviour, IMB constructs and quality of life. The links to the online questionnaire will be sent to all participants automatically via WeChat, and they can fill in the questionnaire on any electronic device, such as smartphones. Questionnaires are checked weekly and reminder messages are sent to participants who have not completed the questionnaires in the required time. If the reminders have been sent twice and ignored twice, a phone call will be made to participants. Participants can receive an online cash transfer of 10 Chinese Yuan (CNY), 20 CNY and 20 CNY (50 CNY in total, which equals approximately 7 US dollars) if they finish the follow-up questionnaires in each of months 3, 6 and 12.

**Table 3:**
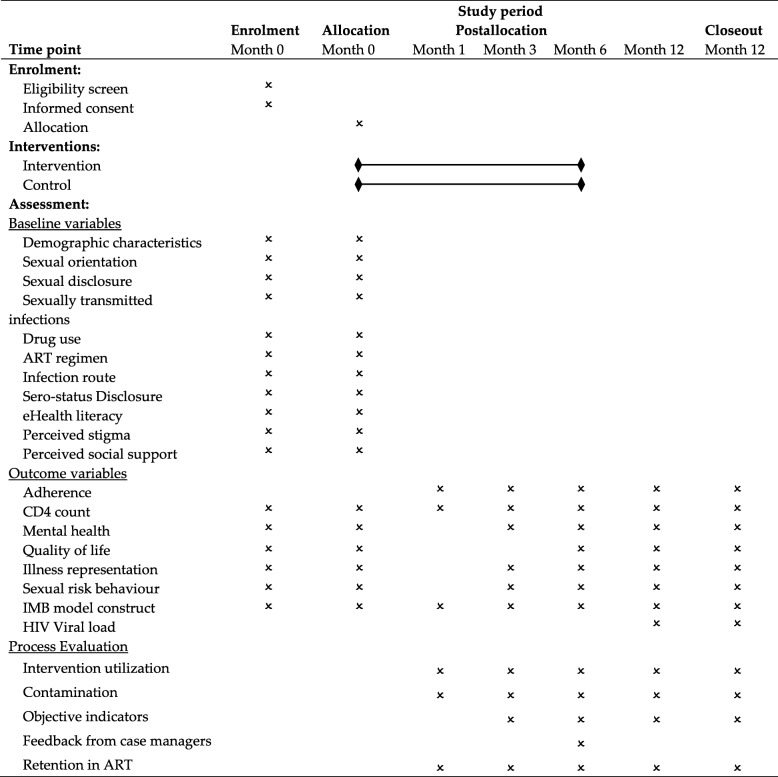
Schedule of enrolment, interventions, and assessments

### Measures

#### Primary outcome

The primary outcome of this study is self-reported ART medication adherence and CD4 count at month six. As literature studies have shown that self-reported adherence is usually positively skewed, mostly due to social desirability bias and recall bias [45], we adopt a composite scale that has high sensitivity [46]. This scale consists of three adherence items measuring medication-taking adherence in the preceding 30 days: 1) a ‘days missed’ item, in which participants are asked the number of days they have missed an ART dose; 2) a ‘rating’ item, in which participants are asked to rate their pill-taking performance; 3) a ‘frequency’ item, in which participants are asked how frequently they took their pills according to instructions. Cronbach’s alpha was 0.86 in the previously reported study using this scale [47]. Moreover, item responses for the three adherence items were linearly transformed to a 0–100 scale and the average scores of all three items are used. A summary of the individual adherence items was calculated as the mean of the three individual items. In this study, we consider anyone scoring below the maximum score on any item as non-adherent (i.e., a combined scale score of < 100). CD4 counts are examined in each physical test and measured by flow cytometer. The months one and three assessments allow for trend evaluation and the month 12 assessments will allow for evaluation of persistent effect of intervention, but these are not the primary outcomes.

#### Secondary outcomes

***HIV-1 RNA*** viral load at month 12 was measured by polymerase chain reaction (PCR).

***Mental health*** status includes anxiety and depression, which are measured by the seven-item Generalized Anxiety Disorder-7 (GAD-7) instrument [48] and nine-item Patient Health Questionnaire-9 (PHQ-9) [49], respectively. Both instruments comprise four-point Likert scales with the responses based on occurrence of symptoms during the last week. In each scale, the sum of all items will be calculated and categorized into different levels of mental health disorder: normal (0–4), mild (5–9), moderate (10–14), and moderate to severe (≥15) [50, 51].

***Quality of life*** is measured by the 31-item HIV adaptation of the World Health Organization Quality of Life scale (WHOQOL-HIV bref) [52], which has shown good reliability and validity among Chinese PLWHA [53]. The scale consists of six domains: physical health, level of independence, psychological health, spirituality, social relations and environmental health. For each domain, a score ranging from 4 to 20 will be calculated, with higher scores denoting better quality of life.

***Illness representation*** is measured by the Brief Illness Perception Questionnaire (B-IPQ), which has been used among PLWHA to evaluate their cognitive and emotional response towards HIV [54]. The B-IPQ comprises the following eight dimensions: consequences, timeline, personal control, treatment control, identity, coherence, concern, and emotional response. Each dimension has only one question, with responses rated on a scale from 0 to 10. A higher score indicates a greater perception of a threat from HIV [55].

***ART-related perception*** is measured by the Life Windows Information-Motivation- Behavioural Skills ART Adherence Questionnaire (LW-IMB-AAD) [56]. The instrument consists of 33 items rated on a 5-point Likert scale, with nine measuring information, ten measuring motivation, and 14 measuring behavioural skills. Summative scores are computed for each aspect, with higher scores reflecting better information, motivation and behavioural skills about adherence [57].

***Sexual risk behaviour*** before and after diagnosis is measured separately using self-constructed items, including numbers of male/female and regular/casual partners, and frequency of condom use. Example items include, ‘How many male partners have you had sex with after diagnosis?’, ‘How many of them are regular (or casual) partners?’ and ‘How often have you used condoms with your regular male partners during the last 3 months?’

#### Process evaluation

In addition, process evaluation indicators are also measured. The main components in process evaluation are contamination (e.g., ‘Have you subscribed to other information resources that provide HIV- or ART-related information?’ and ‘Have you ever consulted the research team’s WeChat account?’) and the usage of mHealth intervention (e.g., ‘Have you ever initiated online communication with case managers/read delivered articles/searched for articles of interest within the WeChat platform?’, ‘What topics have you consulted your case managers about?’, and ‘How frequently do you use this WeChat platform?).

Participants will also be asked to provide an overall comment about the extent to which the platform helps in their daily life. Qualitative interviews will be conducted with the four nurse case managers to investigate the feasibility and acceptability of design and implementation of the intervention. Objective process evaluation indicators will be retrieved from the intervention APP, including the number of times each educational article has been read, the number of enrolled patients for each case managers, the numbers of days when case managers had conversation with patients, and the total numbers of messages sent by case managers. In addition, with the consent of participants, the frequency of key words emerging in the conservation will be counted, to identify the top priority of patients during the initial ART phase.

### Sample size estimation

We will calculate the size of effect that the sample size in our study can detect with 80% power (two-tailed alpha 0.05) with regard to the primary outcome. Based on previous studies conducted among Chinese MSM that newly initiate ART, we estimate that the adherent rate at month six in the control group will be 89% [14]. The target sample size of 600 (300 in each arm) will be powered to detect a smallest between-group difference of 9% in adherent rates, allowing for a 20% attrition rate at month six.

### Data storage and management

All paper documents, including informed consents, service contracts and notebooks of enrolment information will be stored in a locker in the principal investigator’s office at Sun Yat-sen University. Digital data will be retrieved after the study period, stored on a password-protected computer, and deleted permanently from the ‘Trusted Doctor’ server. To preserve confidentiality, all direct personal identifiers will be removed and replaced with a RID in the data files, which will also be protected with a password. Only persons who are part of the research team will have access to the data. Nurses’ APP accounts and investigators’ WeChat accounts are password-protected for exclusive use in this study. No personal information will be documented on the investigator’s WeChat contact; each participant who friends an investigator will be given their RID as a WeChat alias.

### Statistical analysis

#### Baseline comparison

The equivalence between the baseline characteristics of the two arms will be analysed by calculating the standard difference (SDiff). A value > 0.1 will reveal imbalance.

#### Effectiveness of intervention

The primary outcomes will be the prevalence of ART adherence and CD4 counts at month six. For the between-group comparisons, *χ*^*2*^ test will be used for binary outcomes (e.g., ART adherence, mental health disorders, sexual risk behaviours) and *t*-test for continuous outcomes (e.g., CD4 count, quality of life, illness representation, IMB constructs, and HIV viral load). Within-subject analyses will also be conducted, comparing the baseline response to follow-up session responses.

Intervention effects will be performed on the principle of intention-to-treat (ITT), using the data collected from all randomized participants in the analysis. Missing endpoints will be imputed by Multiple-Imputation approach. The General estimating equation (GEE) will be used for repeated measures analyses using SAS 9.0, which allows for the inclusion of all subjects in the analysis regardless of their number of visits. As GEEs require data to be randomly missing, the analysis will be repeated using a subset of participants with complete data. A sensitivity analysis will be conducted to determine the impact of dropout and to evaluate additional longer timeframes (month 12).

#### Intervention mechanism analysis

It has been hypothesized that ART adherence information, motivations, and skills will be improved by the intervention, and will function as mediators of the intervention effect on improving ART adherence and psychosocial health outcomes. Structural equation modelling (SEM) will be adopted to test the applicability of the hypotheses using AMOS 17.0.

## Discussion

To our knowledge, this study is the first to explore the efficacy of mHealth intervention in the case management services targeted at HIV-infected MSM in low-and middle-income countries that incorporate timely online communication between case managers and patients, comprehensive educational articles, supportive service information and hospital-visit reminders.

As there is an unprecedented number of patients on ART after the country-wide scale-up of this therapy-provision, the Chinese CDC issued the ‘Exploration of HIV case management service model’ initiative, requiring case managers to deliver exclusive follow-up services for PLWHA in designated hospitals [58]. Research on the case management of HIV patients is still at an early stage in China, but some evidence already suggests that case management leads to better adherence to treatment and quality of life, and to less risky sexual behaviour [59, 60]. However, Chinese case managers continue to be burdened with exceedingly heavy workloads and restrained by limited policy and financial support [61, 62].

In this scenario, APP-based mHealth interventions have the potential to improve service delivery and positively affect patient outcomes [23]. Smartphones are more portable than computers, and together with their accessibility and intensive daily use by many people, they can enable point-to-point individualised interventions and help reduce some societal and structural barriers faced by stigmatised populations [28]. This study protocol provides a clear and thorough description of an APP-based mHealth intervention and will be used to investigate the effectiveness of mHealth intervention and underlying mechanisms. Findings in this study will provide new insights into HIV-infected MSM patients’ daily struggles of ART adherence and will also suggest how for mHealth services may be delivered in a cost-effective manner at a clinic and national level. There are several strengths of this study. First, the intervention is theory-driven and based on an adherence-targeting framework, IMB theory, which is a widely-adopted theory in mHealth intervention development [26]. Second, extensive elicitation research was conducted and multi-dimensional intervention components were integrated accordingly to tailor health services. Third, this study covers a wide range of measurements, including adherence, the biological parameters of HIV disease-progress (i.e., CD4 count and HIV RNA), mental health outcomes, and quality of life. Fourth, the linkage of an intervention APP and the WeChat platform means that the patients can use this highly accepted platform for daily communication and receiving intervention services, rather than having to download a new ‘ART-treatment’ APP, which patients may be reluctant to do. Fifth, adequate training and supervision is provided to the nurses, including the development of SOP and programme manuals. Moreover, the user experience of participants and the feedback of case managers and the intervention group as well as objective process evaluation indicators will be recorded and analysed, to investigate the feasibility and applicability of the intervention in the future.

There are a few study limitations worth noting. Most importantly, the open study-design means that we cannot mask the intervention, thus participants will be aware that they are part of an ART adherence intervention, which might introduce bias. Although self-reported adherence to ART has notable defects, we have adopted a composite and sensitive measurement scale, using CD4 counts and HIV-RNA levels as objective indicators of efficacy. In addition, we will not be able to control external factors that may confound study results, although we will document some potential confounders (e.g., health information received from other sources or other health programmes occurring in the intervention period) to help with the interpretation of findings.

This trial is among the first efforts to innovatively develop and test the smartphone APP-based case management mHealth intervention in China. Once proven effective, the innovative mHealth service could be integrated into the routine case management of PLWH. This mHealth intervention could also tailored to the patient management service for other chronic conditions. The trial is currently ongoing and the intervention effects will be reported after the data collection.

## Data Availability

The datasets during and/or analyzed during the current study are available from the corresponding author on reasonable request.

## Abbreviations

AIDS: Acquired immune deficiency syndrome
APP: Application
ART: Antiretroviral treatment
CD4: Cluster of differentiation 4
CDC: Centre for disease control and prevention
CNY: Chinese Yuan
GAD: Generalized anxiety disorder
GEE: General estimating equation
HIV: Human immunodeficiency virus
IMB: Information-motivation-behavioural skills
IPQ: Illness perception questionnaire
ITT: Intention-to-treat
LGBT: Lesbian gay bisexual transgender
LW-IMB-AAQ: Life windows information-motivation- behavioural skills art adherence questionnaire
mHealth: mobile health
MSM: Men who have sex with men
NGO: Non-governmental organization
PHQ: Patient Health Questionnaire
PLWHA: People living with HIV/AIDS
PPI: Patient and public involvement
RID: Research identification
SDiff: Standard difference
SEM: Structural equation modelling
SOC: standard-of-care
SOP: Standard operating procedure
SPIRIT: Standard protocol items: recommendations for interventional trials
STD: Sexually transmitted diseases
TasP: Treatment as prevention
WHOQOL: World Health Organization quality of life scale
